# Iron deficiency anemia induced by magnesium overuse: a case report

**DOI:** 10.1186/s13030-019-0159-y

**Published:** 2019-07-30

**Authors:** Hiroshi Sugimoto, Ui Yamada

**Affiliations:** Division of Psychosomatic Medicine, Department of Internal Medicine, St. Luke’s International Hospital, 9-1, Akashi-cho, Chuo-ku, Tokyo, 104-8560 Japan

**Keywords:** Iron deficiency anemia, Magnesium overuse, Anorexia nervosa

## Abstract

**Background:**

Although in vitro studies show that iron absorption can be inhibited by magnesium laxatives such as magnesium oxide, taking oral iron supplements with magnesium laxatives is not considered a clinical problem.

**Case presentation:**

A 28-year-old woman diagnosed with anorexia nervosa who overused magnesium laxatives was admitted to our hospital to evaluate her refractory iron deficiency anemia (IDA), despite having taken oral iron replacement therapy for nine months. She had had amenorrhea for years and her fecal occult blood tests were negative. Furthermore, upper gastrointestinal endoscopy showed no suspected gastroduodenal bleeding or gastroenteritis. We considered her IDA to be induced by malabsorption of iron due to magnesium laxative overuse. Psychoeducational intervention stopped the overuse and oral iron replacement therapy was switched to the intravenous route. During outpatient follow-up, her anemia gradually improved; however, when her magnesium laxative overuse began again, her hemoglobin levels suddenly decreased.

**Conclusions:**

Clinicians should be attentive to the interactions between iron and magnesium laxatives.

## Background

Iron deficiency anemia (IDA) is common in women, especially in pre-menopausal women because of menstrual blood loss. Oral iron replacement therapy is considered the first-line therapy for IDA; however, in vitro studies show that iron absorption can be inhibited by magnesium laxatives, such as magnesium oxide [1]. Nevertheless, taking oral iron supplements with magnesium laxatives is not considered a clinical problem.

## Case presentation

A 28-year-old Japanese woman was admitted to our hospital to evaluate her refractory IDA despite having taken oral iron replacement therapy for nine months. She was diagnosed with anorexia nervosa when she was 15 years old and had begun self-induced vomiting two years previously. One year later, she started to overuse over-the-counter magnesium laxatives instead of doing self-induced vomiting. She took 50 to 200 tablets (16.7 to 66.7 g) of magnesium oxide daily, which is equivalent to approximately 10 to 40 g per day of elemental magnesium. Her hemoglobin level was 11.7 mg/dL about 20 months before she started to overuse magnesium laxatives. Within three months of starting magnesium overuse, her hemoglobin level had decreased to 8.7 mg/dL and IDA was suspected; thus, oral iron replacement therapy with 50 mg of sodium ferrous citrate was started by her primary care physician. Prior to referral at our hospital, she was taking no medicines except for magnesium oxide and sodium ferrous citrate.

On admission, she had persistent general malaise. Her height, weight, and body mass index were 163.5 cm, 32.0 kg, and 12.0 kg/m^2^, respectively. Her laboratory results revealed the following: hemoglobin 5.5 g/dL, mean corpuscular volume (MCV) 59.1 fL, serum iron 8 μg/dL, total iron binding capacity (TIBC) 488 μg/dL, ferritin 13.7 ng/mL, reticulocyte 1.81%, reticulocyte hemoglobin equivalent (RET-He) 16.5 pg, leukocyte count 2.9 × 10^3^ /μL, and platelet count 483 × 10^3^ /μL. Her vitamin B_12_ and folic acid levels were within normal range (577 pg/mL and 5.5 pg/mL, respectively). She had IDA with refractory to long-term iron replacement therapy, and although she had had amenorrhea for years, her fecal occult blood tests (twice) were negative, anti-*Helicobacter pylori* antibody was negative, and there was no acute dietary change, phlebotomy, or excess intake of tannin acid. Furthermore, upper gastrointestinal endoscopy showed no suspected gastroduodenal bleeding or gastroenteritis. We did not perform colonoscopy.

**Fig. 1:**
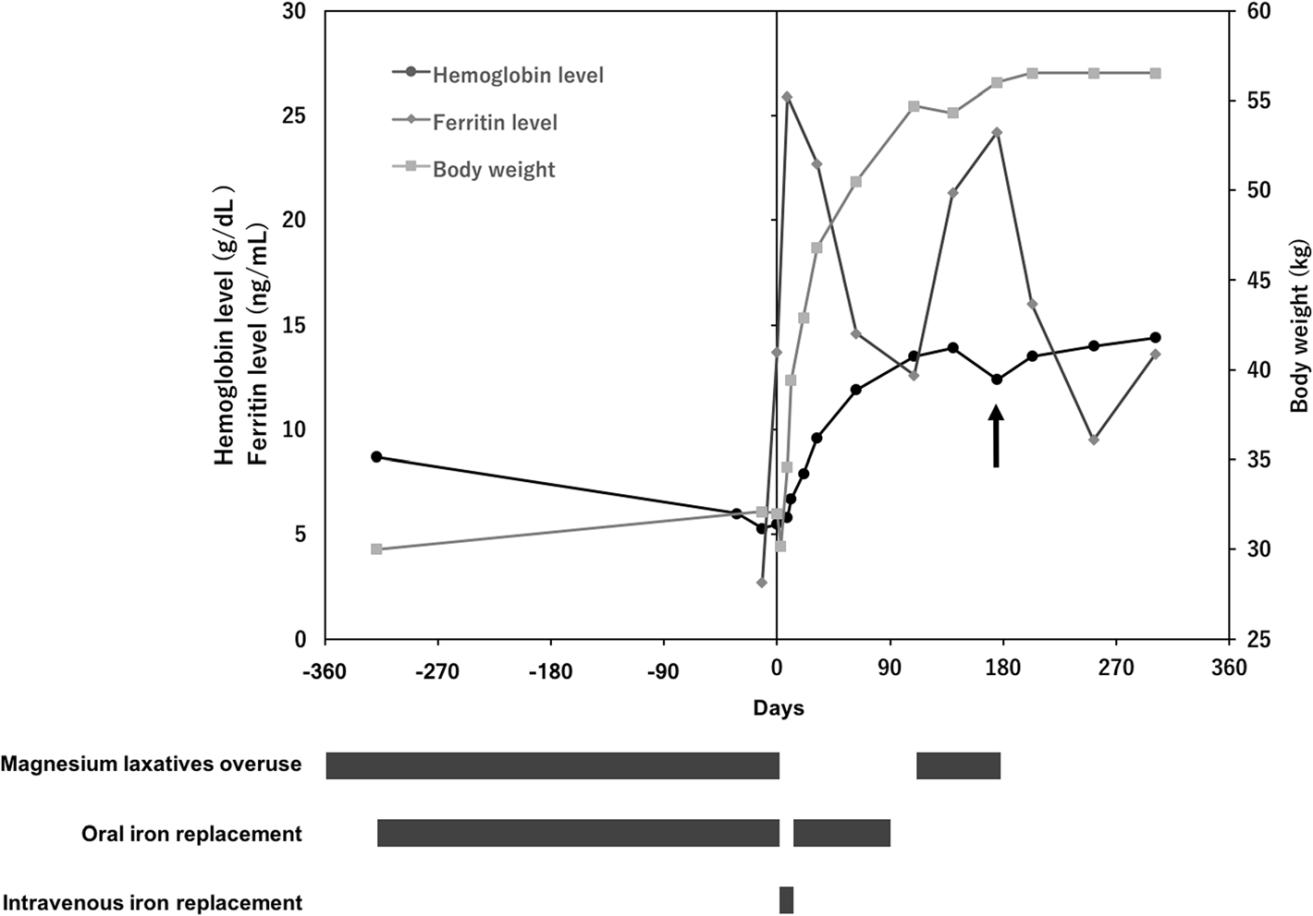
Hemoglobin level, ferritin level, and body weight changes before and after patient admission. At around 180 days, the hemoglobin level unexpectedly decreased when her magnesium laxative overuse began again (arrow)

We considered her IDA to be induced by malabsorption of iron due to magnesium laxative overuse; psychoeducational intervention stopped the overuse and oral iron replacement therapy was switched to 40 mg of intravenous saccharated ferric oxide for 10 days. Additionally, enteral nutrition was provided via a nasogastric tube. After her hemoglobin level increased to 6.7 g/dL, she was discharged with a prescription of 100 mg of oral sodium ferrous citrate. During outpatient follow-up, her anemia gradually improved; however, when her magnesium laxative overuse began again, her hemoglobin levels suddenly decreased, with no obvious bleeding episode, other causes of anemia, or dietary change (Fig. 1).

## Discussion and conclusions

IDA is reported to be rare, with the anemia associated with anorexia nervosa due to menstrual cessation and gelatinous bone marrow transformation [2]. Further follow-up for IDA should be performed, especially for patients with anorexia nervosa.

Magnesium is the most common intracellular divalent cation and is an essential co-factor for enzymatic reactions in the body [3]. The tolerable upper intake level (UL) for supplemental magnesium has been determined to be 350 mg/day by the Food and Nutrition Board. In the present case, the patient’s magnesium intake was 100-fold above the UL. [4] Magnesium oxide is widely used as a laxative or antacid and is available over the counter in many developed countries. It is well known that magnesium laxatives interact with medicines, including oral antibiotics, bisphosphonates, digitalis, and iron supplements [1, 5]. Furthermore, it has been reported that long-term use of acid modifying medications such as proton pump inhibitor, histamine-2 receptor antagonist, and calcium carbonate can cause IDA [6]. However, administering oral iron supplements with magnesium laxatives is not considered to be a clinical problem, especially with sodium ferrous citrate which is less influenced by the gastric pH level. [7, 8]

IDA induced by magnesium carbonate pica has been reported, and significant binding between iron and magnesium carbonate was presumed to be the mechanism of iron malabsorption [9]. An in vitro study that examined the interaction between iron and antacids, including magnesium oxide, revealed that magnesium oxide can cause iron malabsorption by decreased pH and formation of macromolecular polymer, even in the case of sodium ferrous citrate [10]. With a similar mechanism, it was suggested that excessive oral intake of magnesium oxide can also induce IDA, as observed in the present case, which was refractory to oral iron replacement therapy. Therefore, clinicians should be attentive to the interactions between iron and magnesium laxatives, although further investigation will be required to clarify if the normal dosage of magnesium laxatives can induce IDA in usual settings.

## Data Availability

Not applicable.

## Abbreviations

IDA: Iron deficiency anemia
UL: Tolerable upper intake level
